# Cost–benefit analysis of the CoCare intervention to improve medical care in long-term care nursing homes: an analysis based on claims data

**DOI:** 10.1007/s10198-022-01546-7

**Published:** 2022-12-08

**Authors:** Boris A. Brühmann, Klaus Kaier, Rieka von der Warth, Erik Farin-Glattacker

**Affiliations:** 1 Institute of Medical Biometry and Statistics, Section of Health Care Research and Rehabilitation Research (SEVERA), Faculty of Medicine and Medical Center, University of Freiburg, Freiburg, Germany; 2 Institute of Medical Biometry and Statistics, Division Methods in Clinical Epidemiology, Faculty of Medicine and Medical Center, University of Freiburg, Freiburg, Germany

**Keywords:** Claims data, Cost analysis, Secondary data, Propensity score, Complex health intervention, Nursing home residents, I10, H51

## Abstract

**Background:**

Providing adequate medical care to nursing home residents is challenging. Transfers to emergency departments are frequent, although often avoidable. We conducted the complex CoCare intervention with the aim to optimize nursing staff–physician collaboration to reduce avoidable hospital admissions and ambulance transportations, thereby reducing costs.

**Methods:**

This prospective, non-randomized study, based on German insurance data, includes residents in nursing homes. Health care cost from a payer perspective and cost–savings of such a complex intervention were investigated. The utilisation of services after implementation of the intervention was compared with services in previous quarters as well as services in the control group. To compensate for remaining differences in resident characteristics between intervention and control group, a propensity score was determined and adjusted for in the regression analyses.

**Results:**

The study population included 1240 residents in the intervention and 7552 in the control group. Total costs of medical services utilisation were reduced by €468.56 (*p* < 0.001) per resident and quarter in the intervention group. Hospital stays were reduced by 0.08 (*p* = 0.001) and patient transports by 0.19 (*p* = 0.049). This led to 1.66 (*p* < 0.001) avoided hospital days or €621.37 (*p* < 0.001) in costs–savings of inpatient services. More services were billed by general practitioners in the intervention group, which led to additional costs of €97.89 (*p* < 0.001).

**Conclusion:**

The benefits of our intervention clearly exceed its costs. In the intervention group, avoided hospital admissions led to additional outpatient billing. This indicates that such a multifactorial intervention program can be cost-saving and improve medical care in long-term care homes.

**Supplementary Information:**

The online version contains supplementary material available at 10.1007/s10198-022-01546-7.

## Background

Due to changes in fertility and mortality rates during the twentieth century, the share of people aged 65 or older in Germany and most other European countries will increase substantially in the coming decades [1, 2]. Out of this age group, currently, 4% are living in nursing homes (NHs), reaching 18% in the group 85 and older [3]. This leads to 794,917 elderly people in Germany living in NHs in the year 2019, receiving full-time residential care [4]. It is becoming increasingly challenging to provide adequate medical care to nursing home residents (NHRs) [5, 6]. While other countries like the Netherlands or France provide specialized geriatric medical care for NH residents, in Germany, general practitioners (GPs) handle this in addition to their regular workload [7].

Referrals of NHRs to emergency rooms (ERs) are common: in Germany, up to 50% of NHRs are admitted to the hospital at least once a year [8], which is in line with hospitalization rates of 9–58% from previous international research [9]. Such visits can be strenuous for NHRs with unclear benefits for their further health progress. A German study found that hospitalization rates in NHs could be reduced by incentivizing visiting physicians with additional compensation, an approach introduced in Germany in 2016 [10].

According to previous research, a fair amount of these transfers might be unnecessary—a systematic literature review concluded that between 4 and 55% of ER transfers could be considered inappropriate [11]. Similar results are found for Germany where 32 of 100 NHRs were hospitalized for ambulatory-sensitive reasons during a year, which could be avoidable by timely prevention or treatment by outpatient health services [12]. Another recent study found comparable results by analysing claims data, reporting 29.6% potentially avoidable hospitalisations (PAHs) [13]. According to a US study, there are 1.8 ER visits per year for every NHR, of which only 53% did not result in hospitalization [14]. Of course, NHs have a vested interest in ruling out fractures or medical negligence claims in the event of an NHR death [15].

PAHs are defined as events that can be handled in ambulatory care and do not require hospitalization—such cases are categorized as ambulatory care-sensitive conditions (e.g., pneumonia and dehydration) [16, 17]. These events cause a substantial economic burden and strain on health care resources worldwide. In a qualitative study conducted in Germany, patients of two emergency departments stated that in addition to unavailable appointments or having to wait a long time, better time-flexibility, the University Hospital’s quality of care and the availability of multidisciplinary care are reasons to seek medical care in an ER [18]. A study conducted in Portugal showed that a total of €250 million (€2,515 per hospitalization) were associated with avoidable hospital admissions which is corresponding to 6% of the total budget of public hospitals in the country. Nearly 84% of the total cost were direct cost of the hospitalization, whereas lost productivity costs (identified as absenteeism and premature death monetized with the human capital approach) were only estimated to be around €40 million [19]. In England, emergency admissions accounted for 67% of hospital bed days at a cost of £12.5 billion per year [20] and have risen by 42% between 2006 and 2017 [21]. Avoiding ER visits and PAHs wherever possible (by, e.g., improving outpatient care and interdisciplinary collaboration) offers a massive cost–savings potential to health care systems worldwide.

There is a growing awareness of the over- and underutilisation of health care services, not just in the health care sector, politics, and research, but also among the general population [22]. A German Health Technology Assessment attests to underuse and misuse of medical services due to a lack of interprofessional collaboration, communication, and documentation between physicians and nursing staff [23]. Tjia et al. [24] found that both nurse preparedness and physician’s attitudes (e.g., professionalism and responsiveness) can make or break interprofessional communication. The study also illustrates the importance of improving physician attitudes, professionalism, and responsiveness to break such a cycle of nursing staff–physician communication breakdown. In another study, essentials for successful cooperation between physicians and NH care workers included mutual trust, a small number of contact persons, fixed agreements, and regular rounds [25]. A change of the resident’s GP to reduce the number of physicians providing care can also be useful as well as linking up with an outpatient clinic when lacking specialist care.

A political response to this misuse of medical services was to introduce collaboration agreements in 2008 between GPs and NHs in the German Social Insurance Code (Sozialgesetzbuch [SGB] V, § 119b). The law was amended several times until 2019 when a mandatory regulation was added. On July 1, 2016, additional reimbursement for outpatient care physicians when treating NHR was introduced on top of their regular budget. Unfortunately, NHs were still experiencing difficulties with the implementation, since negotiations with GPs and specialists about cooperation have to be conducted independently, leading to a lot of bureaucracy [26]. Combined with the common problem of understaffing in NHs, this most often leads to overtime, which is a well-known problem in Germany at an average of 42 h per nurse per 6 months [27]. So far, these new regulations have not led to the desired success.

Since previous efforts have not yielded the desired results, several interventions in Germany are aiming to improve medical care in the long-term care setting and reduce PAH. Among them are Homern [28], HIOPP-3-iTBX study [29], Careplus [30], interprof ATC [31, 32], SaarPHIR [33], BEVOR study [34], Optimal@NRW [35], NOVELLE [36], and a study in Bavarian NHs [25, 37]. These studies set a variety of priorities: some focus on the optimization of interprofessional collaboration [30–32]. Others focus on hospital admissions and assess reasons for hospital admissions and visits to the ER [28], use a practice-based approach (SaarPHIR, Optimal@NRW) to reduce such hospitalisations [33, 35], or have a technical approach (NOVELLE) of developing interdisciplinary emergency algorithms that provide concrete instructions for particularly frequent and relevant emergencies [36]. A study in Bavarian NHs examined prerequisites for successful collaboration between NHs and physicians [25, 37], and the BEVOR study focuses on the effectiveness of Advance Care Planning (ACP) to improve patient-centered care [34].

Health insurance claims data (i.e., administrative and billing data) offer the potential of furthering available data and analyses inherent to complex health services research, at almost no additional cost [38]. Using claims data from German statutory health insurance companies (SHIs) has become an increasingly popular and important part of health services research [39, 40]. However, only a few of the above-mentioned interventions include claims data in their evaluations [28, 33].

### Overview of the intervention

Our study examines the cost–benefit of an intervention for NHRs in long-term care facilities. The Coordinated medical Care project (CoCare) aims to improve the coordination of medical care in long-term care NHs in Germany by optimizing the collaboration of nursing staff and physicians to reduce the number of avoidable hospital admissions and ambulance transportations. It is based on claims data from all German SHI companies with clients in the study region and covers all relevant sectors: outpatient and inpatient treatments, prescription medication, socioeconomic variables, levels of care, etc. The intervention was developed in cooperation with the Association of Statutory Health Insurance Physicians Baden-Wuerttemberg (KVBW) and the Centre for Geriatric Medicine and Gerontology (ZGGF). A detailed overview of the study was published previously [41].

Intervention elements for physicians included: (a) A team of GPs looked after the NHRs. Weekly on-site visits by GPs took place at fixed times and were joined by nursing staff. Although patients were assigned to their designated GP, GPs were allowed to treat any patient on behalf of another GP and could be reached by phone after office hours. (b) Specialists held regular visits, at least quarterly, coordinated by GPs and accompanied by nursing staff. The residents visited were selected by GPs. Additionally, the project supported positioning of suprapubic catheters in the NH, not only by offering training courses for physicians, but also by providing a portable sonography device for each NH if necessary. (c) The intervention included coordinated medication management to expand on usual medical care. Medication plans were written by GPs and monitored quarterly. For issues known to lead to frequent hospitalization of patients, structured preventive measures were established and supported by checklists and action guidelines, developed by geriatricians. (d) In the intervention group, individual medical services administered were reimbursed according to a project-specific compensation plan.

The project also intended to improve communication and collaboration between physicians and nursing staff, by appointing study coordinators (“CoCare coordinators”) at each participating NH as designated points of contact for physicians. CoCare coordinators were in charge of tasks, such as documentation, preparation, and follow-up of on-site physician visits, etc. Additionally, structured processes were facilitated between physicians and nursing staff, such as standard operating procedures for unplanned events (e.g., managing a crisis) and coordination of a physician consult to prevent hospitalization. Treatment procedures (e.g., regarding pain) were structured and developed to involve all specialists and GPs.

We hypothesized that the implementation of CoCare reduces the total cost compared to the control group, which received usual care. Our secondary hypotheses are that the number of hospitalisations, days in hospital, and ambulance transports as well as the costs of hospital stays and ambulance transports decrease following implementation.

## Methods

The evaluation of this complex intervention is part of a study focused on improving the coordination of medical care in long-term care NHs in Germany. In January 2018, the KVBW started the CoCare project in Baden-Wuerttemberg, Germany. Baden-Wuerttemberg is a federal state with 11.1 million inhabitants in the south of Germany. The CHEERS checklist was used as reporting guide [42].

### Sample/study subjects

#### Nursing homes/general practitioners

**Fig. 1:**
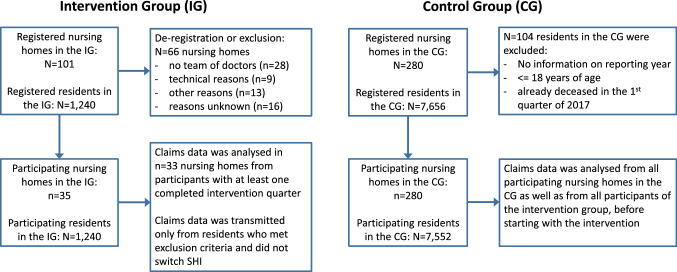
Recruitment of nursing homes

The intervention was administered in 35 NHs concentrated in the counties of the administrative districts of Stuttgart, Karlsruhe, and Freiburg. Claims data from *n* = 33 NHs were analysed from participants with at least one completed intervention quarter. The 280 NHs forming the control group were recruited from the administrative district of Tübingen, which was chosen to mirror the intervention districts in number of physicians, inhabitants, and similarity in counties. Each group did include urban and rural districts to ensure that the results could be generalizable to regions with different structural conditions (e.g., availability of medical care, long-term care NH structure). SHI companies were asked to provide data from NHRs in the control group that were as comparable as possible to those in the intervention group. Control group residents had to be in an NH for at least 90 days and have had at least one of the following nursing home-related fee schedule positions (GOP) billed during the intervention period: 99980, 37102, 37100, 37105, and 37113. Regarding quantity, each SHI selected suitable participants at random until the same percentage as in the intervention group was reached. The number of recruited NHs is displayed below (Fig. 1). The control group mentioned throughout the manuscript additionally includes data from all intervention participants before starting with the intervention.

#### Eligibility criteria and recruitment

NHs that met the following criteria were eligible to participate as a study site for the intervention group and were invited by representatives of the KVBW:

- Willingness to install a secure Internet connection that enables the use of a digital documentation system named CoCare Cockpit.
- Collaboration with a team of GPs participating in the study
- Authorization according to Article 72 of Volume XI of the Social Insurance Code (Elftes Buch Sozialgesetzbuch—SGB XI) [43]

Eligibility criteria for control group NHs were limited to authorization according to Article 72 of Volume XI of the Social Insurance Code. GPs who wanted to participate in the intervention group had to be willing to team up with other GPs.

NHs and GPs were contacted and briefed about the project by the KVBW. NHs which met the criteria were invited to join the study by the KVBW. Based on their location, the NHs were assigned to either the intervention or control group.

#### Residents

The CoCare program was offered to all residents who resided in a participating NH, independent of membership in a specific SHI. Exclusion criteria included dementia and having lived at the facility for less than 3 months. Residents that switched SHI during the study phase were excluded from the study and no data were provided from the SHI regarding these participants. The study included *N* = 1240 residents in the intervention group and *N* = 7656 participants in the control group. In total, *n* = 104 residents in the control group were excluded from the analyses. Reasons included: no available information on the reporting year, being younger than 18 years or the residents were already deceased in the 1st quarter of 2017.

### Study design

This complex intervention is part of a prospective, unblended cluster, non-randomized, controlled study conducted in long-term care NHs in Germany.

### Data collection and measures

#### Observation period

Each participant was observed for a period of 12 months before starting the intervention (year 2017) and a maximum of 21 months after the start of the intervention (January 2018 through September 2019). The individual starting point of each participant depended on the moment the resident decided to participate in the study. Data from NHRs with incoming and outgoing treatment, which exceeded the observation period, were transmitted completely, if possible.

#### Data collection

Data were collected from each resident, which included insured person pseudonym, NH pseudonym, number of days residing in the NH, data on sociodemographics (sex, year of birth, and death), health-related variables (level of care), intervention/control group, and service use and costs (inpatient care, NH care, and ambulatory care). For inpatient care, we had access to data on the treatment (dates of admission and discharge, type of hospital, and the amount reimbursed). For care conducted in the NH, we could access data on change in level of care and the costs incurred. The SHIs received information from the KVBW about patients that declared their consent for data processing within the evaluation, as well as a list with pseudonyms of patients. The data were linked from the different external sources by the evaluating institute (SEVERA) with the help of an individual identification code. Data were provided by the following SHIs: AOK BW, KKH, DAK, BARMER, IKK classic, TK, and BKK-Landesverband Süd as the umbrella organization of employer-sponsored health insurance funds.

#### Intervention costs

All treatment-related costs and costs for the intervention program are measured from an insurer perspective. All reimbursements by the payer (SHIs) in the intervention period are represented. Costs for all treatments consisted of direct costs for inpatient care, NH care, and ambulatory care. Services not covered by their insurance plan (e.g., co-payments for medication) were paid for by NHRs and not included in this analysis. Indirect costs or costs for informal care are of minor importance in NHRs [44]. Intervention start-up costs (e.g., costs of research and development of intervention materials) are excluded, so that the intervention is evaluated as if operating under steady-state conditions. All amounts are expressed in € (EUR).

At the individual resident level, the following cost indicators were used per quarter for the observation periods before and during the intervention:

1. Total cost of hospitalizations
2. Total cost of outpatient billings by the primary care physician
3. Total cost of outpatient billings by the specialist physician
4. Total cost of ambulance transports
5. Total cost of prescribed medications
6. Total cost of prescribed assistive technology (e.g., hearing aids) [45]
7. Total cost of prescribed non-mental health therapy services (e.g., occupational or physical therapy)
8. Total cost of university hospital outpatient clinics.

Complementary to the Uniform Assessment Standard (Einheitlicher Bewertungsmaßstab), a project-specific compensation plan for additional medical project services was applied. The plan included all items listed in Supplemental Table 1 and could only be billed by GPs and specialists who participated in the intervention group. Within this framework, physicians were compensated for individual medical services administered to NHRs, training services, coordinative activities, after-hours availability by phone, and activities preventing hospitalization. These fees were included in outpatient billings by the primary care physician.

### Data analysis

For the cost–benefit analysis, cost indicators (1–8) were analysed on the patient level quarterly (1st quarter 2017 to 3rd quarter 2019). Only data from complete quarters were taken into account. Through the analysis strategy we chose, intervention effects can be determined the same way as in a stepped-wedge design. The utilisation of services after the start of the intervention was compared with the utilisation of services before the start of the intervention as well as with the utilisation of services in the control group. The control group NHs are interpreted as clusters without transition to the intervention phase. In contrast to a stepped-wedge design, the start and location of the intervention was not assigned randomly. Therefore, potential differences in resident characteristics between the intervention and control groups were accounted for using a propensity score adjustment. A propensity score is the probability that a study participant with certain observed baseline values will receive the intervention and thus enables the control of group differences that can arise in non-randomized allocation. In detail, a propensity score was calculated from the resident characteristics age, sex, and care level (at the beginning of the year 2017).

This propensity score was added as a continuous covariate in a two-part regression model with application of cluster-robust standard errors at the NH level to take into account the multilevel structure of the data. This analysis strategy was prespecified in the study protocol [41] and the results of this analysis regarding the endpoint total costs are shown in Supplemental Fig. 1 (base case analysis). As a first sensitivity analysis, the analysis was repeated using a regression adjustment approach: age, sex, and care level are added as continuous (age) and categorical covariates (sex, care level) instead of the propensity score, while all other specifications were identical (see Supplemental Fig. 1, sensitivity analysis 1). As a second and third sensitivity analysis, time trends are accounted for. When analysing studies with a stepped-wedge design, it is generally recommended to incorporate time either as a continuous or categorical fixed effect [46]. Therefore, we repeated the propensity score adjustment approach with the additional inclusion of time (in quarters) as continuous (see Supplemental Fig. 1, sensitivity analysis 2) or categorical (see Supplemental Fig. 1, sensitivity analysis 3) fixed effects. Last but not least, we tried to account for the existence of multiple records on the patient level. Unfortunately, the incorporation of random intercepts is not possible using the applied two-part regression model [47, 48]. We therefore applied a linear mixed model. Although linear mixed models are at disadvantage to account for the right skewness common in cost data, the simplicity of interpretation of the results is a clear advantage. As sensitivity analysis 4, we applied a linear mixed model with NH level and the patient ID as random intercepts. Furthermore, this linear mixed model includes age, sex, and care level and time as continuous (age, time) and categorical (sex, care level) covariates. See Supplemental Fig. 1 (sensitivity analysis 4) and Supplemental Table 2 for details.

The adjusted values mentioned below are values that arise if intervention and control group participants were equal concerning their propensity score. Unadjusted values presented in the Supplemental Table 3 are simple, unadjusted differences between the control and intervention group (or between residents in the intervention NHs before versus after the intervention). The adjusted values are required to test our CoCare program in a scientifically sound way. Therefore, all significance tests refer to the adjusted values.

In a next step, the above-mentioned cost indicators 1 to 8 were aggregated at resident level for intervention and control group. The resulting sum corresponded to the total cost of medical service utilisation of a NHR.

Data sets were collected in a database (MS SQL Server 2017, running Windows Server 2016), computed using SQL and Microsoft Excel 2016 (Microsoft, Redmond, WA, USA) and analysed using the Stata 16 program (StataCorp, College Station, TX, USA) [49].

## Results

### Demographic and resident characteristics

The characteristics of the study population (*N* = 8792) at the beginning of the year 2017 are shown in Table 1. The intervention and control group included data from *n* = 1240 and *n* = 7552 NRHs, respectively. About 65% of the participants in the intervention group were female (control group: 71%), on average 79 years old (control group: 84 years) and mainly insured with the AOK.

**Table 1:** Baseline characteristics

	Intervention group (*N* = 1240)	Control group (N = 7552)	*p* Value*
**Nursing homes**			
Number of nursing homes	33	280	
**Resident characteristics**			
Age in years, mean (SD)	78.93 (12.09)	83.85 (10.38)	< 0.001
Sex (female), *n* (%)	807 (65.10%)	5385 (71.30%)	< 0.001
Level of care**, *n* **(%)**			
0	232 (18.71%)	837 (11.08%)	
I	16 (1.29%)	25 (0.33%)	
II	205 (16.53%)	982 (13.00%)	
III	321 (25.89%)	2,056 (27.22%)	
IV	328 (26.45%)	2,375 (31.45%)	
V	138 (11.13%)	1,277 (16.91%)	
**Residents per health insurance company,** ***n*** **(%)**			
AOK	731 (59.00%)	4,545 (60.20%)	
BARMER	141 (11.40%)	862 (11.40%)	
BKK	76 (6.10%)	504 (6.70%)	
DAK	123 (9.90%)	857 (11.30%)	
IKK Classic	75 (6.00%)	433 (5.70%)	
KKH	40 (3.20%)	2 (0.00%)	
TK	54 (4.40%)	349 (4.60%)	
**Number of assessed quarters of health care utilization**			
	13,052	63,076	

*SD* Standard deviation

*Significance test between the intervention and control group (two-tailed unpaired *t* test for age; two-tailed Chi-square test for sex)

**Level of care = formal categorization of the amount of long-term care a person requires; higher levels indicate greater care requirements

### Costs

**Fig. 2:**
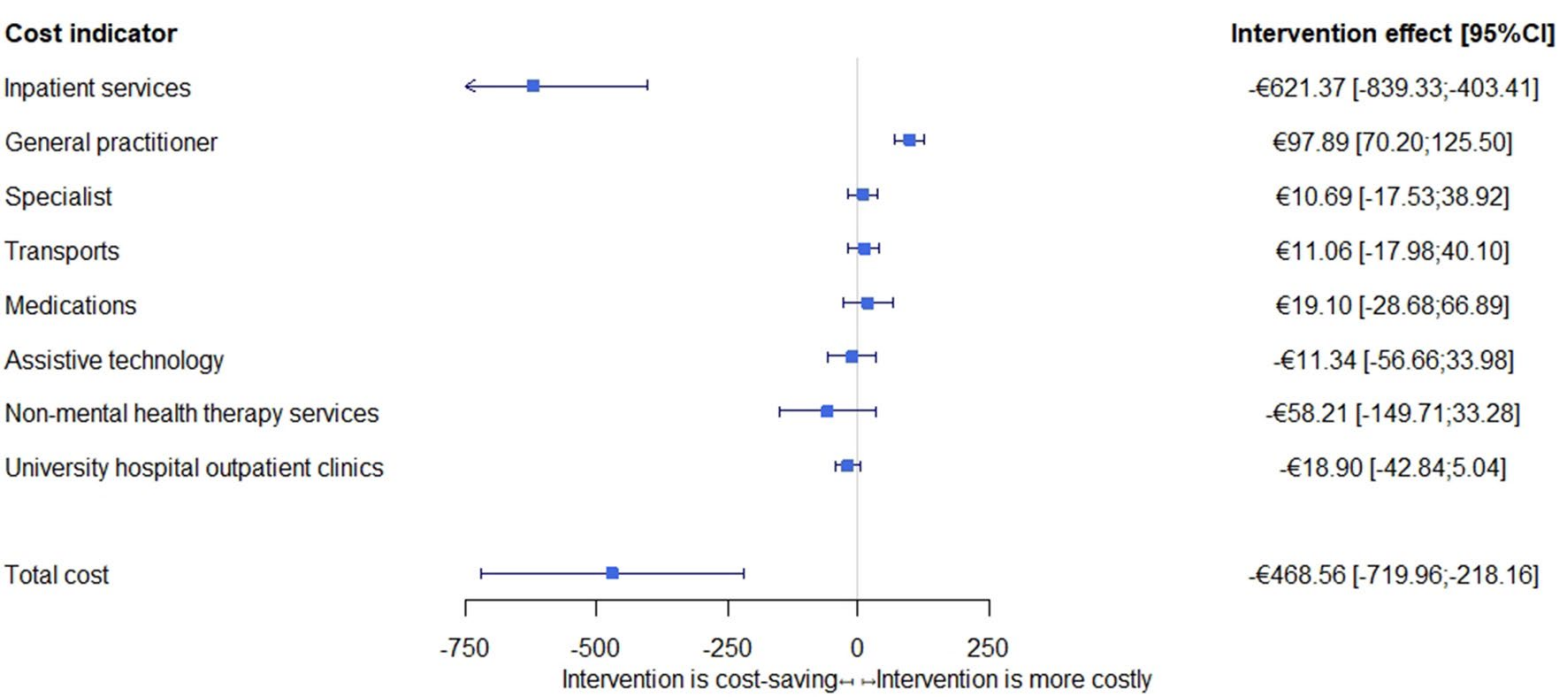
Difference of costs of medical services’ utilisation between intervention and control group

As shown in Fig. 2, the total cost of medical services’ utilisation in the intervention group is reduced by €468.56 (*p* < 0.001) per resident and quarter, with reduced inpatient services cost accounting for the savings despite the increased spending on additional GP services.

Results also show a reduction in hospital stays and patient transports in the intervention group. Per resident and quarter 0.08 (*p* = 0.001) fewer hospital stays are observed (Table 2), corresponding to 1.66 (*p* < 0.001) avoided hospital days per resident per quarter or €621.37 (*p* < 0.001) in savings of inpatient services. The intervention group reduction in patient transports amounts to 0.19 (*p* = 0.049) fewer transports per resident per quarter. A reduction in patient transport or university outpatient clinic costs cannot be confirmed in the intervention group due to lack of significance.

**Table 2:** Adjusted intervention effects during the intervention period

	Effect^a^	*p* Value	95%CI	
**Inpatient services**				
Number of stays	− 0.08	0.001**	− 0.12	− 0.03
Number of hospital days	− 1.66	0.000***	− 2.13	− 1.19
Cost	− €621.37	0.000***	− €839.33	− €403.41
**Outpatient medical service**				
General practitioner—cost incurred	€97.89	0.000***	€70.20	€125.50
Specialist—cost incurred	€10.69	0.458	− €17.53	€38.92
**Hospital transports**				
Number of transports	− 0.19	0.049*	− 0.39	< − 0.01
Cost of transports	€11.06	0.456	− €17.98	€40.10
**Medication**				
Cost of medicines	€19.10	0.433	− €28.68	€66.89
**Assistive technology**				
Cost of assistive products	− €11.34	0.624	− €56.66	€33.98
**Non-mental health therapy services**				
Cost of services	− €58.21	0.212	− €149.71	€33.28
**University hospital outpatient clinics**				
Cost (including outpatient surgeries)	− €18.90	0.122	− €42.84	€5.04
**Total cost**				
Total cost of medical services utilisation	− €468.56	0.000***	− €718.96	− €218.16

^a^Due to the choice of analysis strategy, these effects can be interpreted as causal intervention effects (costs saved or additionally incurred by the intervention/service utilisation per patient and quarter). They refer to the comparison of the intervention group (Post) with the intervention group (Pre) and simultaneously with the control group and are on a quarterly basis

*p < 0.05

**p < 0.01

***p < 0.001

More services per resident were billed by a GP per quarter in the intervention group than in the control group, which led to additional costs of €97.89 (*p* < 0.001) per quarter. The services billed by specialists increased in the intervention group after introducing the intervention per resident and quarter compared to the control group, but no significant difference in cost could be found. A further reduction in physician-related cost regarding medication, assistive technology, or non-mental health therapy services cannot be confirmed due to lack of significance. Further results regarding individual unadjusted costs are presented in Supplemental Table 3.

## Discussion

This analysis evaluates the cost and benefit of a complex intervention focused on improving the coordination of medical care in long-term care NHs in Germany. The project aimed to optimize the collaboration of nursing staff and physicians to reduce avoidable hospital admissions and ambulance transportations, thereby reducing costs when compared to usual care. Results show that the provision of such a multifactorial intervention program is cost-saving for the SHI.

The analysis highlights an advantageous cost–benefit ratio by reducing the total costs of medical service utilisation by €468.56 per NHR and quarter. The cost–savings due to the reduction in hospital stays thus exceed the increased cost of outpatient care by GPs and specialists as a result of the intervention. The expansion of medical care as well as improved communication and coordination between physicians and nursing staff can be regarded as an essential quality indicator of the intervention, irrespective of the costs, and represents a plausible intervention effect.

A comparison of our results with existing literature is difficult, since as of yet no similar interventions with published results of claims data from German SHIs exist. Comparable studies in NHs report only fragments of cost or focus on specific illnesses or items such as hospitalization and have not published any results yet [33, 35]. However, data from international studies evaluating similar interventions to reduce hospitalisations or costs also showed promising results [50–52]. These interventions focused more on a nurse-led model, than the practitioner-led model in CoCare with its emphasis on communication. Results showed that the interventions were in general more effective compared to usual care and that costs as well as hospital admission could be reduced. All studies focused their interventions on nurses, which shows that every NH should build on its internal resources by training experienced nurses to prevent adverse events such as unnecessary hospital admissions. This is similar to CoCare which appointed nurses as CoCare coordinators who were the link between GPs and residents and played an important role for its success. These results support the idea that such interventions can have a positive impact on hospital admissions despite their costs. For stronger evidential support of its effectiveness, such interventions should, however, be evaluated in randomized controlled studies.

A study focusing on overall hospital stays found a reduction of such due to the introduction of additional reimbursement for outpatient care physicians [10]. Similar results can be seen in our study with a reduction of the number of hospital stays and hospital days, which reduced the cost of inpatient services by €621.37. It therefore seems to be an effective tool to reduce PAH admissions in the NH sector, as well as costs. A difference worth mentioning is, however, that Kümpel et al. [10] talk about the reimbursement plan introduced in Germany in 2016 on top of the regular budgets of outpatient care physicians. In our study, an additional reimbursement plan to that introduced in 2016 rewarded physicians further for coming to the NH more often, and for being able to be contacted out-of-hours. This also helped solve the problem mentioned by the authors that physicians (emergency services excluded) generally cannot be reached out-of-hours (at night or during the weekend).

The intervention, with its regularly conducted rounds, leads to expanded care of the NHRs as GPs are more accessible and visit their NHs more often (also additionally upon request). This is reflected in the increased outpatient medical services by GPs leading to additional costs of €97.89. NHRs might face access barriers to specialist care and older people in need of long-term care often have a lower medical specialist utilisation in most medical specialities [53, 54]. Many of them suffer from frailty or cognitive impairment, which often limits their ability to organize adequate health care and visit a specialist practice, especially if the distance is perceived to be long. With the help of weekly visits by GPs, our intervention tried to break such barriers and enable older people to see at least a GP regularly and receive referrals to specialists as necessary.

NHRs in our study were more often than usual treated outpatient than inpatient, as nursing staff could consult the NHRs GP or substitute more easily and weekly visits were part of the intervention. This contrasts beliefs commonly expressed in the literature that NHs have an incentive to have their residents hospitalized rather than calling a physician, especially when they expect the stay to be short term [55]. Therefore, an aim of the intervention was for GPs to collaborate with other GPs, so that such problems could be addressed promptly during the GPs’ or his substitute weekly visits. Preparations for these visits were made by the CoCare coordinators of the NHs to ensure that all arising problems could be addressed as early as possible.

Compared to Kümpel et al. [10], we found a slightly more prominent effect regarding the number of ambulance transports. Transports in our NHs were reduced significantly by 0.19 (*p* < 0.049) per NHR and quarter. This may seem like a small number, but it results in a significant reduction of hospitalization costs and spares NHRs one or two transports over their years at the facility.

A qualitative study with focus group interviews assessing the perceived barriers and facilitators of the implementation of CoCare by stakeholders (nursing staff, GPs, and GP’s assistants) found six themes in respect of the implementation: understaffing, bureaucracy, complexity of the intervention, structural barriers, financial compensation, communication, and collaboration [56]. Barriers, such as understaffing and complexity, are well known in the literature [27, 56]. CoCare, however, provided a good structure to overcome these two barriers, while others will probably dissolve after the intervention is implemented into routine care (e.g., bureaucracy). While not all parts of the CoCare program were perceived as essential in the focus group interviews (a shared patient file created in CoCare Cockpit, sonography device). Certain tools such as regularly scheduled medical rounds or extended availability helped to provide a solid framework to facilitate interprofessional communication, which can also be seen in our results in an increased utilisation of GPs. Participants in the focus groups also perceived CoCare as potentially effective for the prevention of unnecessary hospitalizations of NHRs, which is reflected in our reduced number of hospitalizations. It remains to be seen if analyses of ongoing studies show similar results.

Our study indicates that this program has an advantageous cost–benefit ratio. The intervention effect concerning the total cost of medical service utilisation includes a total saving of €468.56 per NHR and quarter. This suggests cost–savings for the German system of SHI by providing a multifactorial intervention program.

### Strengths and limitations

Our analysis has two essential strengths: first, a large and rich data set on residents in an NH population, including information from *n* = 1240 NHRs participating in the intervention group and *n* = 7552 NHRs in the control group. This allows the study to be adequately powered to demonstrate a cost reduction applicable to other NHs in Germany. Second, the incorporation of claims data from all SHIs in the region provides an adequate basis for deriving representative estimates. Analyses are based on data from a total of seven SHIs or health insurance fund associations in the state of Baden-Wuerttemberg. By including all relevant SHIs of the federal state, it was possible to solve one of the biggest challenges for health care scientists in Germany [38], which minimizes the chance of a selection bias. Therefore, a special feature of the data is the possibility to evaluate the service and prescription data per case (without reference to individuals) and perform a cross-sector analysis.

In interpreting our findings, some shortcomings have to be taken into consideration. First, no randomization was possible, since this is a study in a real-world setting. We determined a propensity score from the resident characteristics age, sex, and care level in the first quarter of 2017, which was adjusted for in the regression analyses, to compensate for remaining differences in resident characteristics between the intervention and control groups. This method is commonly used to address selection bias [40]. Nevertheless, biases in the estimates of the differences in costs due to unobserved variables (e.g., certain morbidity conditions) cannot be ruled out. This also includes factors that were measured right before the intervention, which could not be included in the risk adjustment process, since the intervention was rolled out over a longer time period.

Second, the dataset incorporated a large number of minimal costs, which is common in cost data, violating the assumption of a normal distributed dependent variable in regression models. We chose a two-part regression model with a logistic and generalised linear model with log-link and negative binomial distribution (or gamma distribution in the case of costs) to account for the right skewness.

Third, we could not account for costs being generated by the intervention (acquisition of a transportable sonography device and laptop, and compensation for the NHs for participating in the study and providing CoCare coordinators) or outside the patient-level cost settlements. Costs generated by the provision of a laptop can be ignored, as NHs were bound to introduce a digital documentation system in their facility with the introduction of a new quality and inspection system for full inpatient care on the 1st of October 2019 [57]. It is similar with the received compensation by the NHs for implementing the intervention which can be disregarded, since these costs would not occur under normal circumstances. This leaves only costs outside of the patient-level cost settlements and the transportable sonography device. Both of those costs were impossible to include in the analyses as they could not be billed by the SHI and could therefore not be implemented or distributed evenly among participating NHRs and quarters.

## Conclusion

The additional outpatient care of NH patients, often triggered by improved remuneration, can be regarded as an essential quality indicator of the intervention, irrespective of the costs, and represents a plausible intervention effect. The benefits of the intervention (avoided hospital admissions) clearly exceed the costs (additional outpatient billings). If implemented correctly, such a multifactorial intervention program can be cost-saving and improve medical care in long-term care NHs. The intervention seems to be a useful tool to reduce PAHs as well as costs in the NH sector. It remains to be seen if other ongoing studies show similar results.

### Supplementary Information

Below is the link to the electronic supplementary material.

Supplementary file1 Supplemental Table 1: Project-specific fee schedule (PDF 71 KB)

Supplementary file2 Supplemental Table 2: Multivariable linear mixed model regarding the endpoint total costs (PDF 41 KB)

Supplementary file3 Supplemental Table 3: Unadjusted costs in intervention and control group before and after the introduction of the intervention (PDF 45 KB)

Supplementary file4 Supplemental Figure 1: Sensitivity analyses regarding the endpoint total costs (PDF 45 KB)

## Data Availability

The datasets underlying this article are owned by the statutory health insurances (AOK BW, KKH, DAK, BARMER, IKK classic, TK, and BKK-Landesverband Süd as the umbrella organization of employer-sponsored health insurance funds). Since public deposition of the data would breach ethical and legal compliances, data are only available upon request. To request the data, please contact the SEVERA (erik.farin@uniklinik-freiburg.de). To fulfill the legal requirements to obtain data, researchers must obtain a permission for a specific research question from the German Federal (Social) Insurance Office. Additionally, researchers must negotiate a contract with all the statutory health insurances regarding data access. The licensee is permitted to use the data for the purpose of the research proposal within their company, exclusively. Here, company is defined as an economical unit. Licensees are not allowed to pass the data to a third party, or to create software or data bases with the exception of scientific publications. Moreover, the study has to be approved by the data protection officers of all the participating statutory health insurances, the applying research institute, and the evaluating institute.

## Abbreviations

AOK BW: Allgemeine Ortskrankenkasse Baden-Wuerttemberg, statutory health insurance
BARMER: Statutory health insurance
BKK-Landesverband Süd: Landesverband der Betriebskrankenkassen Süd, as the umbrella organization of employer-sponsored health insurance funds
CoCare: Coordinated medical care
DAK: Deutsche Angestellten-Krankenkasse, statutory health insurance
ER: Emergency room
GOP: Fee schedule position (Gebührenordnungsposition)
GP: General practitioner
IKK Classic: Statutory health insurance
KKH: Kaufmännische Krankenkasse, statutory health insurance
KVBW: Association of statutory health insurance physicians Baden-Wuerttemberg
NH: Nursing home
NHR: Nursing home resident
SHI: Statutory health insurance
TK: Techniker Krankenkasse, statutory health insurance
ZGGF: Centre for Geriatric Medicine and Gerontology
