# Supplementary material for: Cost–benefit analysis of the CoCare intervention to improve medical care in long-term care nursing homes: an analysis based on claims data
Source: Eur J Health Econ. 2022 Dec 8;24(8):1343–55. doi: 10.1007/s10198-022-01546-7 (PMC10533715; doi:10.1007/s10198-022-01546-7)
Supplement: Supplementary file 1 — Supplementary file1 Supplemental Table 1: Project-specific fee schedule (PDF 71 KB) [file 10198_2022_1546_MOESM1_ESM.pdf]

Supplemental Table 1: Project-specific fee schedule[1]

Code	Description	Fee
99930<br><br>99930A	Surcharge for joint GP-patient rounds on-site<br>*Minimum requirement(s):*<br>- Participation in GP patient rounds of one or more nursing home residents, at a previously agreed upon and jointly scheduled time, attended by an appointed member of nursing staff<br>- Selection of residents that require specialty care referrals in coordination with nursing home<br>Billable once per visited resident and visit, max. once per week, nursing home, and resident.	€10.00<br><br>starting at 2nd patient: €5.00
99931<br><br>99931A	Surcharge for joint specialty physician-patient rounds on-site<br>*Minimum requirement(s):*<br>- Participation in specialty physician-patient rounds of one or more nursing home residents, at a previously agreed upon and jointly scheduled time, attended by an appointed member of nursing staff<br>Billable once per visited resident and visit, max. once per week, nursing home, and resident.	€15.00<br><br>starting at 2nd patient: €5.00
99932	Interdisciplinary, indication-specific case conference with duration minimum of 15 minutes per patient<br>*Minimum requirement(s):*<br>- Participation in an interdisciplinary case conference on-site to coordinate for the following indications between nursing home, physicians, and–if applicable–relatives or other conference participants: Transition from curative to palliative care, challenging behaviors (Behavioral and psychological symptoms of dementia (BPSD)/neuropsychiatric symptoms).<br>Case conferences are billable once per quarter and patient. Billable only by attending GPs and speciality physicians. Duration minimum of 15 minutes. Billable a maximum of two times per completed 15 minutes per session and patient.	€25.00
99933	Quarterly meetings with a duration minimum of 45 minutes<br>*Minimum requirement(s):*<br>- GP participation in quarterly meeting with nursing staff<br>Billable once per nursing home and quarter, if necessary by phone, max. 3 times per calendar year and not billable in quarters where yearly meetings take place. Duration minimum: 45 minutes.	€90.00
99934	Yearly meetings with a duration minimum of 45 minutes<br>*Minimum requirement(s):*<br>- GP or specialty physician participation in a joint annual meeting with all attending physicians and nursing staff.<br>Billable once per nursing home and calendar year, if necessary by phone, not billable in the same quarter as quarterly meeting.	€90.00
99935/<br>99946	Flat fee for additional medical documentation<br>*Minimum requirement(s):*<br>- Entries to the joint digital medical records file (CoCare Cockpit), either on-site or at physician's office.<br>Billable once per physician, patient and quarter.	€40.00

Cont.: Annexe 1: Project-specific fee schedule[1]

CPT	Description	Fee
99936	Surcharge for medication check and coordinated medication management<br>*Minimum requirement(s):*<br>- Review and, if indicated, adjustment of all medication by attending GP using a comprehensive medication plan (including all specialty physician prescriptions and OTC)<br>- Coordination with nursing staff<br>*Optional, additional service(s):*<br>- Use of electronic medication plans<br>- Coordination with other prescribing physicians<br>Billable every second quarter, starting at three active ingredients	€10.00
99937	Participation in trainings<br>*Minimum requirement(s):*<br>- Joint participation of GP and nursing home coordinator in one-day training course offered by ZGGF<br>Billable once per GP	€100.00
99938	Surcharge for extended availability<br>*Minimum requirement(s):*<br>- Phone availability and on-call times extended until 9 p.m. Monday through Friday for calls from nursing facility.<br>*Optional, additional service(s):*<br>- On-call availability for several nursing facilities<br>Billable once per on-call day and physician	€50.00
99939	Surcharge for phone consultation with nursing facility during extended availability hours<br>*Minimum requirement(s):*<br>- Phone consultation during extended availability hours (after 7 p.m. or on weekends)<br>*Optional, additional service(s):*<br>- Evaluate indications for hospitalization<br>Billable for each phone consultation; not billable in addition to urgent visit (99941)	€8.00
99940	Surcharge for consultation with emergency (services) physicians<br>*Minimum requirement(s):*<br>- Phone consultation between GP and emergency (services) physician to coordinate care or prevent hospital admission<br>Billable for each phone consultation; not billable in addition to urgent visit (99941)	€10.00
99941	Surcharge for urgent GP visit<br>*Minimum requirement(s):*<br>- Same-day visit by GP upon request<br>*Optional, additional service(s):*<br>- Evaluate indication for hospitalization<br>Billable once per patient and day visited	€15.00

Cont.: Annexe 1: Project-specific fee schedule[1]

CPT	Description	Fee
99942	Surcharge for urgent urologist visit<br>*Minimum requirement(s):*<br>- Same-day visit by urologist upon request<br>*Optional, additional service(s):*<br>- Evaluate indication for hospitalization<br>Billable once per patient and day visited	€15.00
99943	Surcharge for change or removal of suprapubic catheters by GP or urologist on-site<br>*Minimum requirement(s):*<br>- Change or removal of suprapubic catheter by GP at the nursing facility<br>- Coordination of catheter management<br>*Optional, additional service(s):*<br>- Use of sonography device provided as part of the project<br>- Collaboration with nursing staff<br>Billable per patient and catheter change/removal	€25.00
99944	Surcharge for insertion of suprapubic catheter by urologist on-site<br>*Minimum requirement(s):*<br>- Insertion of suprapubic catheter at the nursing facility by urologist<br>- Coordination of catheter management<br>*Optional, additional service(s):*<br>- Use of sonography device provided as part of the project<br>- Collaboration with nursing staff<br>Billable per patient and catheter insertion	€50.00
99945	Subsidy for establishing a connection to SNK via KV-SafeNet<br>*Minimum requirement(s):*<br>- Establishing a new connection from office computer (or private computer used for work) to SNK via KV-SafeNet<br>Billable once per GP	€100.00

[1] Fee schedule has been amended as necessary throughout the project, this being the most recently used version.
