# Supplementary material for: Cost–benefit analysis of the CoCare intervention to improve medical care in long-term care nursing homes: an analysis based on claims data
Source: Eur J Health Econ. 2022 Dec 8;24(8):1343–55. doi: 10.1007/s10198-022-01546-7 (PMC10533715; doi:10.1007/s10198-022-01546-7)
Supplement: Supplementary file 2 — Supplementary file2 Supplemental Table 2: Multivariable linear mixed model regarding the endpoint total costs (PDF 41 KB) [file 10198_2022_1546_MOESM2_ESM.pdf]

Supplemental Table 2: Multivariable linear mixed model regarding the endpoint total costs

	Coefficient	p value	95%CI	
**Intervention**	-442.19	<0.001	-659.64	-224.74
**Female sex**	-564.31	<0.001	-701.78	-426.85
**Age (in years)**	-61.74	<0.001	-67.79	-55.69
**Care level (Reference = 0)**				
1	-415.60	0.332	-1254.42	423.22
2	-492.77	<0.001	-729.20	-256.34
3	-346.55	0.001	-556.56	-136.54
4	-249.40	0.020	-459.23	-39.57
5	-220.38	0.068	-456.80	16.04
**Quarter (1st quarter 2017 = 0)**	40.67	<0.001	28.21	53.13
**Constant**	8168.37	<0.001	7655.77	8680.96
