# Supplementary material for: Cost–benefit analysis of the CoCare intervention to improve medical care in long-term care nursing homes: an analysis based on claims data
Source: Eur J Health Econ. 2022 Dec 8;24(8):1343–55. doi: 10.1007/s10198-022-01546-7 (PMC10533715; doi:10.1007/s10198-022-01546-7)
Supplement: Supplementary file 3 — Supplementary file3 Supplemental Table 3: Unadjusted costs in intervention and control group before and after the introduction of the intervention (PDF 45 KB) [file 10198_2022_1546_MOESM3_ESM.pdf]

Supplemental Table 3: Unadjusted costs in intervention and control group before and after the introduction of the intervention

	**Control group** (N=63,076 patient quarters)		**Intervention group (Pre)** (N=9,689 patient quarters)		**Intervention group (Post)** (N=3,363 patient quarters)	
	Mean	SD	Mean	SD	Mean	SD
**Inpatient services**						
Number of stays	0.23	0.81	0.24	0.62	0.18	0.53
Number of hospital days	2.38	9.75	2.96	13.76	1.43	6.22
Cost	€1,045.83	€4,706.99	€1,266.52	€6,270.99	€680.27	€2,792.72
**Outpatient medical service**						
General practitioner - Cost incurred	€199.14	€303.84	€248.96	€340.32	€313.79	€292.68
Specialist - Cost incurred	€88.45	€438.15	€104.78	€439.12	€118.20	€535.37
**Hospital transports**						
Number of transports	0.89	3.52	0.69	2.01	0.74	2.70
Cost of transports	€135.27	€417.35	€124.21	€381.73	€149.91	€505.79
**Medication**						
Cost of medicines	€435.17	€1,263.07	€421.40	€732.50	€499.23	€702.49
**Assistive technology**						
Cost of assistive products	€243.31	€1,214.53	€207.32	€857.44	€223.20	€621.62
**Non-mental health therapy services**						
Cost of services	€385.28	€1,053.48	€259.20	€774.95	€331.69	€863.26
**University hospital outpatient clinics**						
Cost (including outpatient surgeries)	€38.04	€125.60	€15.35	€71.89	€22.93	€82.56
**Total cost**						
Total cost of medical services utilisation	€2,570.50	€5,471.63	€2,647.75	€6,710.62	€2,339.23	€3,655.27

SD = Standard deviation
