# Supplementary figures and images for: Cost–benefit analysis of the CoCare intervention to improve medical care in long-term care nursing homes: an analysis based on claims data
Source: Eur J Health Econ. 2022 Dec 8;24(8):1343–55. doi: 10.1007/s10198-022-01546-7 (PMC10533715; doi:10.1007/s10198-022-01546-7)

Supplemental Figure 1: Sensitivity analyses regarding the endpoint total costs

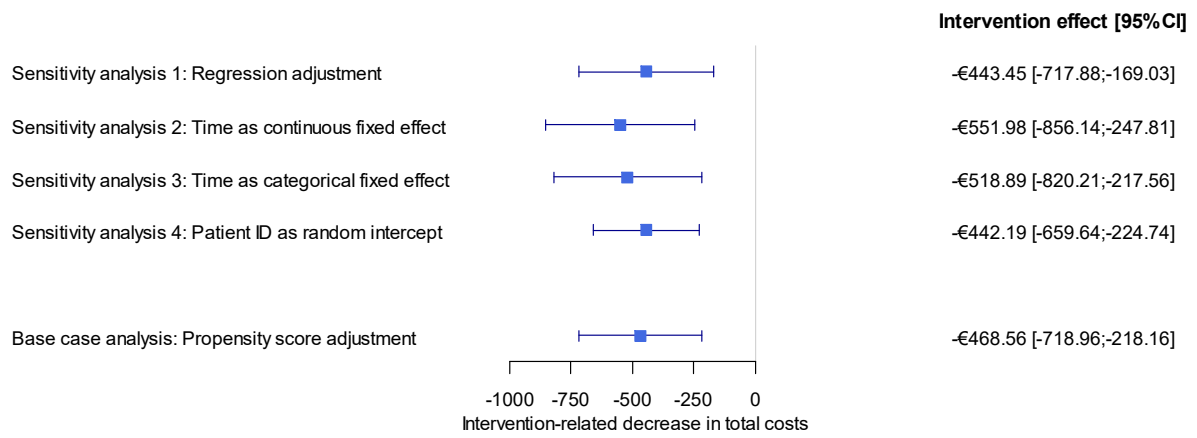

Supplement: Supplementary file 4 — Supplementary file4 Supplemental Figure 1: Sensitivity analyses regarding the endpoint total costs (PDF 45 KB) [file 10198_2022_1546_MOESM4_ESM.pdf]
